# Facilitators and barriers to vaccination uptake in pregnancy: A qualitative systematic review

**DOI:** 10.1371/journal.pone.0298407

**Published:** 2024-04-19

**Authors:** Mohammad S. Razai, Rania Mansour, Pahalavi Ravindran, Samuel Freeman, Charlotte Mason-Apps, Joan Morris, Azeem Majeed, Michael Ussher, Sally Hargreaves, Pippa Oakeshott

**Affiliations:** 1 Population Health Research Institute, St George’s, University of London, London, United Kingdom; 2 University Hospitals of Leicester NHS Trust, Leicester, United Kingdom; 3 University Hospitals Sussex NHS Foundation Trust, Sussex, United Kingdom; 4 Department of Primary Care and Public Health, Imperial College London, London, United Kingdom; 5 Institute for Social Marketing and Health, University of Stirling, Stirling, United Kingdom; 6 The Migrant Health Research Group, Institute for Infection and Immunity, St George’s, University of London, London, United Kingdom; Universidade Federal de Minas Gerais, BRAZIL

## Abstract

**Introduction:**

Vaccination during pregnancy protects both the mother and the foetus from vaccine-preventable diseases. However, uptake of the recommended vaccines (influenza, pertussis, COVID-19) by pregnant women remains low in Europe and the USA. Understanding the reasons for this is crucial to inform strategies to increase vaccination rates in pregnant women. This qualitative systematic review aimed to identify the barriers and facilitators to vaccination against influenza, pertussis/whooping cough and COVID-19 during pregnancy and identify possible strategies to increase vaccination rates.

**Methods:**

We conducted a comprehensive search of electronic databases, including Medline, PsycINFO, CINAHL, Web of Science, WHO database, Embase and grey literature to identify qualitative studies that explored barriers and facilitators to vaccine uptake among pregnant women (PROSPERO CRD42023399488). The search was limited to studies published between 2012 and 2022 conducted in high-income countries with established vaccination programmes during pregnancy. Studies were thematically analysed and underwent quality assessment using the Joanna Briggs Institute validated critical appraisal tool for qualitative research.

**Results:**

Out of 2681 articles screened, 28 studies (n = 1573 participants) were eligible for inclusion. Five overarching themes emerged relating to personal, provider and systemic factors. Barriers to vaccine uptake included concerns about vaccine safety and efficacy, lack of knowledge about vaccines’ benefits and necessity, fear of adverse effects on the foetus or mother and low perception of disease severity. Facilitators included recommendations from trusted healthcare providers, easy access to vaccination, clear communication on the benefits and safety of vaccination, and positive social influences from family and friends. Strategies for increasing vaccination uptake included strong and proactive vaccine recommendations by trusted healthcare professionals, provision of vaccines during routine antenatal care, and clear and consistent communication about vaccines addressing pregnant women’s concerns.

**Conclusion:**

This review highlights the need for interventions that address the identified barriers to vaccine uptake among pregnant women. Recommendation from a healthcare provider can play a significant role in promoting vaccine uptake, as can clear risk/benefit communication and convenient access to vaccination. Addressing concerns about vaccine safety and providing accurate information about vaccines is also important.

## Introduction

Pregnant women are at increased risk from viral pathogens due to physiological and immunological changes during pregnancy [1]. Unvaccinated pregnant women with SARS-CoV-2 or influenza infections are at higher risk of severe disease, hospitalisation, complications and death than vaccinated women [2–8]. Similarly, pertussis/whooping cough infection primarily affects infants, leading to high rates of hospitalisation and death [9–11].

Multiple studies have shown that vaccination confers very high levels of protection against severe disease outcomes in pregnancy from COVID-19 [12–15] and influenza [17, 18].

One recent study showed that vaccine effectiveness against severe COVID-19 complications for all pregnant women was 48% (95% CI 22–65) with a complete regimen and 76% (47–89) after a booster [19]. Similarly, inactivated influenza vaccination in pregnancy lowered confirmed influenza cases by 63% in infants and prevented about a third of febrile respiratory illnesses in both mothers and young infants [17]. Most importantly, vaccination is safe in pregnancy [3, 4, 20–23,]. Maternal pertussis immunisation protects the infant through the passive and active transfer of maternal antibodies [24, 25]. Therefore, all three vaccines (influenza, pertussis and COVID-19) are strongly recommended during pregnancy [26]. The U.S. Centers for Disease Control and Prevention (CDC) has advised seasonal influenza vaccination for pregnant women since 1997, followed by Australia in 2009 and the UK in 2010. Pertussis vaccination, recommended between 16–32 weeks of gestation for each pregnancy, was added to the CDC’s guidelines in 2010. The UK and Australia adopted this recommendation in 2012 and 2015, respectively [27]. More recently, in the UK, pregnant women have been advised to have an autumn COVID-19 booster vaccine [28].

Despite this, low vaccine uptake among pregnant women was reported for 2022 in both the USA (pertussis 44%; COVID-19 ≥ 1 dose 61%; influenza 50%) [29] and the UK (COVID-19 two doses 51%; pertussis 60%; influenza 30%) [30–32]. Furthermore, across high-income countries, high levels of vaccine hesitancy (delay in acceptance or refusal of vaccines despite availability of vaccine services) [33] have been reported for the influenza [34], pertussis [35, 36] and COVID-19 vaccines [3, 22, 38–48]. Vaccine hesitancy is a significant contributor to low vaccine uptake and is listed as one of the top ten global health threats by the World Health Organization [49]. This phenomenon is multifaceted depending on temporal, geographical and sociodemographic contexts [49]. Risk factors for lower vaccine uptake in pregnancy include younger age [3, 22, 37], greater socioeconomic deprivation [3, 22, 37] and minority ethnicities, mainly Black and Latino communities. [3, 42, 47, 48]. Additionally, migrant groups have lower vaccination rates [38, 50–52].

Studies on factors influencing vaccination decision-making during pregnancy suggest that healthcare professional (HCP) recommendations and beliefs about vaccine safety and efficacy are key drivers of vaccination uptake [53–55]. However, the impact of HCP recommendations could be diminished by factors such as belief in vaccine-induced harm, particularly for novel vaccines like COVID-19 [54, 56, 57]. Additional influences included personal sentiments, rumours, trust, and cultural values [34]. Recent qualitative research has identified belief in conspiracy theories and misinformation as obstacles to vaccine uptake among pregnant women [56]. Previous systematic reviews have mainly included quantitative methodologies [53] and may have focused less on the complexity, context, nuance and meaning of vaccinations in pregnancy. Our study aims to fill this knowledge gap.

### Aims

We conducted a systematic review and thematic synthesis of qualitative studies investigating the facilitators and barriers to vaccine uptake during pregnancy in high-income settings with established vaccination programmes. Additionally, while there have been best practice reports, government guidelines, and opinion articles (including by authors MSR, PO, AM, SH, MU) addressing vaccine hesitancy in the general population [49, 58–62], there are few proven interventions designed for vaccine hesitancy in pregnancy. The aim of this systematic review was to identify facilitators and barriers to vaccination for three recommended vaccines (influenza, pertussis, COVID-19) during pregnancy, and to explore strategies for enhancing uptake.

## Methods

### Data sources and search strategy

A systematic literature review of qualitative studies on vaccination in pregnancy was conducted according to the Preferred Reporting Items for Systematic Reviews and Meta-Analyses (PRISMA) [63]. The review protocol was registered on PROSPERO (International Prospective Register of Systematic Reviews; CRD42023399488). Although the protocol included several research questions, this systematic review specifically concentrates on the facilitators and barriers to vaccination during pregnancy using qualitative data. (Other questions have been addressed in separate analyses [55]).

On 15^th^ December 2022, we searched peer-reviewed and grey literature across multiple databases using keywords (S1 Table). We searched Embase, Web of Science, Oxford Academic Journals, PubMed NIH, Clinical Trials, China CDC, CDC reports, and the WHO COVID-19 global literature database for COVID-19 literature [64]. Non-COVID-19 literature was searched using Embase, CINAHL, PsycINFO, and Medline. The searches were complemented with relevant grey literature by scanning key institutional websites (e.g., Royal College of Obstetrics & Gynaecology and UK Health Security Agency) and Google Scholar by hand search. The search strategy (see PROSPERO protocol) was guided by the key domains and determinants of vaccine hesitancy based on our Five Cs of vaccination: confidence, complacency, convenience, communication and context [65]. The Five C’s model is used to understand the psychological and behavioural determinants influencing vaccine hesitancy or acceptance. This model is particularly useful in public health settings and is comparable to the WHO’s Behavioural and Social Drivers (BeSD) of Vaccination framework [66]. In addition, the review evaluated the facilitators and barriers to vaccine uptake in pregnancy, drawing from routine vaccination and COVID-19 literature. The included studies were quality-assessed using PRISMA guidelines and Joanna Briggs Institute (JBI) validated Critical Appraisal Tool for Qualitative Research [67].

### Eligibility criteria

We assessed the papers against the inclusion criteria (**Table 1**). We included qualitative and mixed-method study designs that used data collection methods such as interviews or focus groups with pregnant women, to examine barriers and facilitators to vaccination in pregnancy in high-income settings. We excluded studies with only healthcare providers and no pregnant women. Studies with recently post-partum women (pregnant within the past year) were included if they enquired about vaccine uptake during pregnancy.

**Table 1:** Inclusion criteria.

• Location: High-income settings as defined by the World Bank • Publication years: from 2012–2022 • Population: pregnant women or pregnant within the past year • Vaccinations: influenza, pertussis, COVID-19 • Outcome measures: pregnant women’s vaccination status and intention to vaccinate • Study type: qualitative and mixed method • Papers: related to the outcome measures including grey literature (i.e., government guidelines, preprints).

Data screening, extraction, and quality assessment

Title and abstract screening of the initial search was performed independently by four investigators. Once the abstracts were regarded as relevant, the entire paper was reviewed against the inclusion criteria. Both the title and abstract screening, as well as the full-text review, were completed in duplicate for around 70% of the papers. Any discrepancies were resolved by discussion between authors.

Predefined data were extracted from each study. This included first author; year of publication; study design, location and date; vaccine of interest (i.e., COVID-19, influenza, pertussis); sample size; basic demographic details of participants (ethnicity, age and educational attainment); gestational age. Core findings related to facilitators and barriers to vaccination during pregnancy were captured from relevant studies and synthesised. Two reviewers (MSR and RM) used the JBI Critical Appraisal Tool for Qualitative Research [67] to independently assess the risk of bias in the included studies. The tool consists of a series of questions that researchers use to critically appraise each study included in the review. The questions include the study’s design, sampling, data collection and analysis, researcher reflexivity, and the researchers’ interpretation of the data (**S2 Table**). Studies are scored out of ten, with scores of eight to ten considered high quality, five to seven medium, and one to four low. Discrepancies were resolved through discussion, and where a decision could not be reached, a third reviewer (PO) arbitrated. To allow a comprehensive overview, no studies were excluded based on quality assessment.

### Data synthesis and analysis

We conducted a qualitative analysis of all relevant articles, utilising the thematic synthesis approach described by Thomas and Harden (2008). This approach was selected to facilitate the development of analytical themes that extend beyond primary studies and to provide new insights and implications for policy, practice, and future research. Two authors (MSR and RM) reviewed each relevant article and conducted line-by-line coding of the results sections, capturing first-order concepts (participants’ interpretations of their experience) and second-order concepts (authors’ interpretation of participants’ experience) using Microsoft Excel and Word. We employed an inductive approach to coding without preconceived assumptions regarding how codes should be defined and structured. The resulting codes were then compared across studies to identify specific facilitators and barriers to vaccination in pregnancy. These were subsequently grouped and organised under descriptive themes. The barriers, facilitators, and descriptive themes were further compared and discussed across studies, with this iterative process informing the development of analytical themes. The researchers also sought to identify key strategies to increase vaccination during pregnancy. Each reviewer (MSR and RM) independently coded about 50% of the studies, followed by a discussion to agree on the codes and resolve any discrepancies.

## Results

### Study characteristics

**Fig 1:**
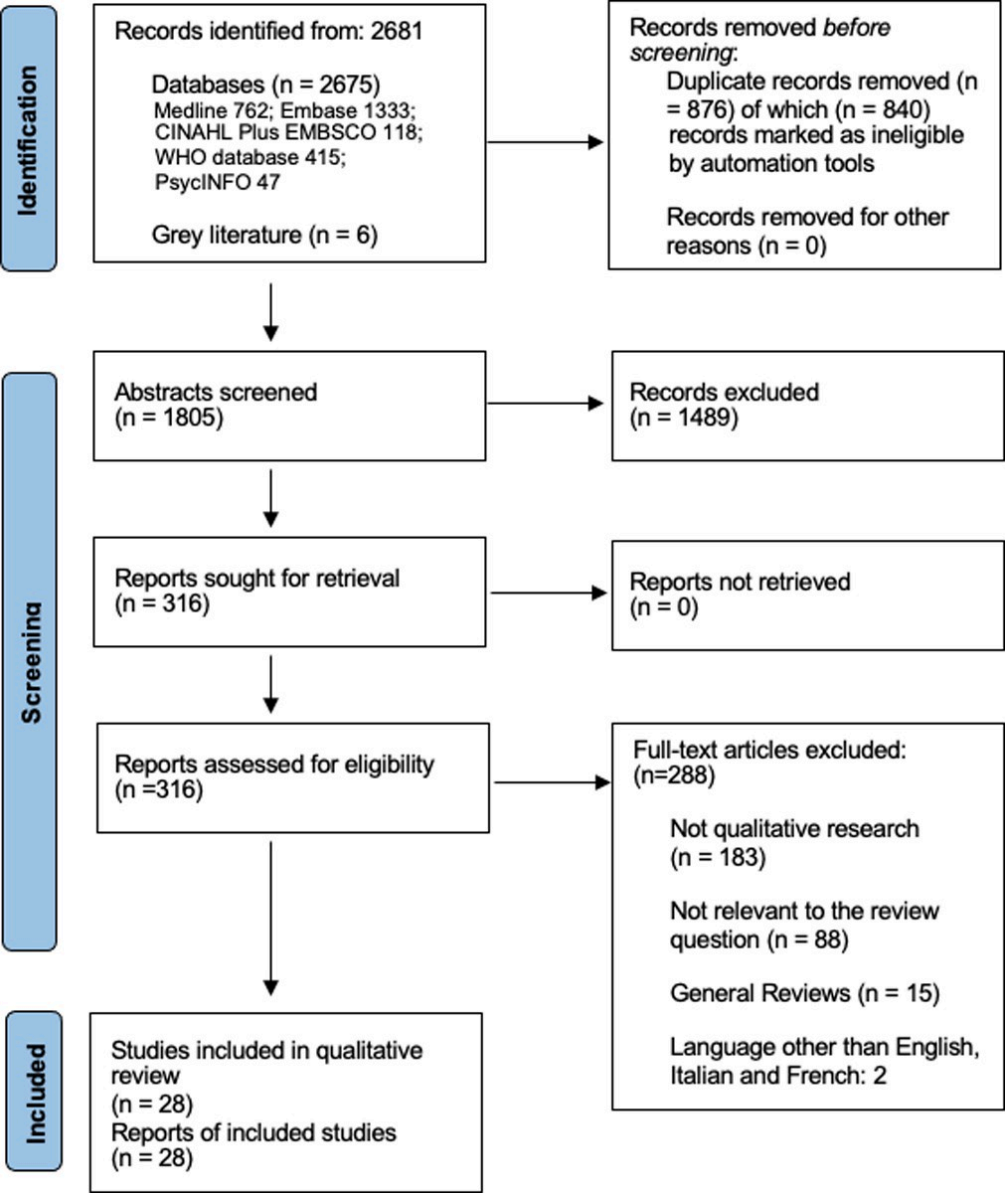
Systematic review PRISMA flow diagram.

**Fig 1** shows the PRISMA flow diagram [63]. There was a total of 28 included studies (n = 1573 participants). A summary of the descriptive characteristics of these articles is shown in **Table 2**. The sample size ranged from 7 to 441, with 15 (54%) studies having less than 30 participants. Most studies (19/28, 68%) reported ethnicity, with the majority of participants being of White ethnicity. Only seven studies (25%) reported participants’ educational attainment. Among these, around a third of the participants (98/268, 37%) had completed a university education. In addition, four studies reported on participants’ income, while two reported on their socioeconomic status. The median publication year was 2018 (range = 2012–2022). Most studies (21/28, 75%) only used qualitative methodology, and the rest were mixed methods. The included studies utilised a blend of semi-structured interviews, interview questionnaires, focus groups, or a combination of these methods for data collection. The UK was the country with the most studies (9/28, 32%), followed by Australia and New Zealand (7/28, 25%) and the USA (4/28, 14%). The majority of studies (22/28, 78%) of vaccination in pregnancy focused on influenza and pertussis rather than COVID-19. The studies were evenly divided between hospital and community settings, with 14 conducted in hospital settings, 13 conducted in community settings, and one study that did not specify its setting. Half the studies (14/28, 50%) were considered high quality, and the rest were medium quality.

**Table 2:** Characteristics of included studies (N = 28).

	Year	Author	Country	Setting	Vaccine of Inquiry	Design, Approach, Data Collection & Analysis	Sample Size	Age, Years	Ethnicity of Participants	Education Level	Income Level	Gestation of Participants	Quality Score
**1**	2012	Schindler et al.	Switzerland	Not stated	Influenza	**•** Design: Qualitative **•** Approach: Not reported **•** Collection: Semi- structured interview **•** Analysis: Coded using ATLAS.ti	29	Mean: 34 Range: 19–40	Not reported	Not reported	Not reported	Women were within 3–5 days of giving birth	6/10
**2**	2013	Meharry et al.	USA	Hospital	Influenza	**•** Design: Qualitative **•** Approach: Not reported **•** Collection: Written questionnaire, semi- structured interview **•** Analysis: Content analysis	60	Mean: 32 Range: 18–45	24 American white 6 Black 12 Hispanic 13 European 4 Asian 1 Australian	13 school or less 11 some college 16 university degree 20 graduate degree	15 ≤ $50,000 9 > $50,000 7 not disclosed	Women were either in their 3rd trimester, or new mothers on the postpartum unit.	9/10
**3**	2014	Marsh et al.	USA	Hospital	Influenza	**•** Design: Qualitative **•** Approach: Grounded theory **•** Collection: Semi- structured interviews **•** Analysis: Thematic analysis	21	Mean: 24.5 Range: 19–39	21 African American	Not reported	Not reported	Gestational age 8–36 weeks	6/10
**4**	2014	Collins et al.	Australia	Hospital (tertiary)	Influenza and Pertussis	**•** Design: Qualitative **•** Approach: Not reported **•** Collection: Semi- structured interviews **•** Analysis: Iterative thematic analysis techniques	17	18 and over	14 Australian, 2 Indonesian 1 Sri Lankan	Not reported	Not reported	Any woman in their 1st-3rd trimester	7/10
**5**	2015	Donaldson et al.	England	Antenatal Care Clinic	Pertussis	**•** Design: Mixed **•** Approach: Not reported **•** Collection: Questionnaire **•** Analysis: Thematic content analysis of free text	200	Mean: 31.4 Range: 18+	40 Asian 37 Black 88 White 9 Mixed 19 Other 7 Did not report	Not reported	Not reported	At least 27 weeks pregnant. Average gestation 32 weeks.	6/10
**6**	2016	Yuen et al.	Hong Kong	Hospital	Influenza	**•** Design: Qualitative **•** Approach: Not reported **•** Collection: Semi- structured Interview **•** Analysis: 2-step thematic analysis process, manual data management strategy	32	25–29 (n = 9) 30–34 (n = 11) >35 (n = 12)	Not reported	21 no university degree 11 university degree	6 < median income 26 median income or greater	Women had to have recently given birth to a live new-born	8/10
**7**	2015	Wiley et al.	Australia	Hospital Antenatal clinics at 2 tertiary hospitals and 1 rural hospital	Pertussis and influenza	**•** Design: Qualitative **•** Approach: Grounded theory **•** Collection: Semi- structured interviews and iterative analysis cycles **•** Analysis: Line-by-line coding, thematic analysis using NVivo	20	Not reported	Not reported	Not reported	Not reported	Pregnant woman at any gestational age	8/10
**8**	2015	O’Grady et al.	Australia	Community	Influenza	**•** Design: Mixed • Approach: Not reported **•** Collection: Yarning circles (focus groups) guided by semi- structured narrative inquiry **•** Analysis: Thematic analysis	7	Range: 21–34	7 Aboriginal and/or Torres Strait Islander	18 some school 19 school graduate 13 post school qualification 4 no response	Not reported	Women were more than 28 weeks’ gestation or less than 16 weeks post birth.	9/10
**9**	2017	Winslade et al.	UK	Baby clinics run by health visiting services	Pertussis	**•** Design: Qualitative **•** Approach: Not reported **•** Collection: Semi- structured face-to-face interviews. **•** Analysis: thematic analysis.	42	Late adolescence to mid-forties’	’Majority were White British’	Not reported	Not reported	Mothers with babies born post-UK’s 2012 vaccination programme	7/10
**10**	2016	Gauld et al.	New Zealand	Community pharmacy	Pertussis	**•** Design: Qualitative **•** Approach: Framework **•** Collection: Structured questionnaire **•** Analysis: Deductive analysis using Mindmeister	37	Range: 18–43	22 NZ European 4 Māori 6 Pacific Island 3 Indian 3 Other	Not reported	Not reported	Women with a child aged 1 year or younger	7/10
**11**	2016	Bettinger et al.	Canada	Hospital	Influenza	• Design: Mixed **•** Approach: Not reported **•** Collection: Focus group **•** Analysis: Direct content analysis, using the Health Belief Model & Theory of Planned Behaviour	34	20–29 (n = 7) 30–39 (n = 24) 40+ (n = 3)	Not reported	3 trade college 9 high school 22 university	7 < $35,000 6 $35,000–75,000 15 > $75 000 6 not disclosed	Median gestational age of participants was 31 weeks, with a range of 10 to 40 weeks.	8/10
**12**	2018	O’Shea et al.	Ireland	Hospital (maternity)	Influenza and pertussis	**•** Design: Qualitative **•** Approach: Not reported **•** Collection: Semi- structured interviews **•** Analysis: thematic analysis using MAXQDA software	17	Mena: 33 Range: 23–44	12 Irish 1 Polish 1 Nigerian 1 Spanish 1 Maltese 1 Pakistani	Not reported	Not reported	Women were within 1 month of delivery.	8/10
**13**	2018	Maisa et al.	UK (Northern Ireland)	Community	Influenza and pertussis	**•** Design: Qualitative **•** Approach: IPA **•** Collection: 3 focus groups, 1 in-depth interview. **•** Analysis: Thematic analysis using NVivo 10; Applied COREQ criteria	16	Range: 18–44	Not reported	Not reported	Not reported	Women were at least 16 weeks pregnant	7/10
**14**	2019	Wilson et al.	UK	Community	Influenza and Pertussis	**•** Design: Qualitative **•** Approach: Not reported **•** Collection: Interviews on NHS maternity care experiences & 16-week check video recordings **•** Analysis: Thematic analysis of vaccination discussions; deductive & inductive coding using NVivo V.11	40	Range: 18 to 41	White British, Black British, Japanese, Nigerian, German, Australian, Chinese, South African, Somalian, Orthodox Jewish, Pakistani British, Brazilian, Turkish, Norwegian, Italian, Lithuanian	Not reported	Not reported	Pregnant at time of interview or recently pregnant	7/10
**15**	2020	de Munter et al.	Netherlands	Community	Pertussis	**•** Design: Qualitative **•** Approach: Grounded theory **•** Collection: Structured interview questionnaire; focus groups **•** Analysis: Managed by ATLAS.ti 8 for interviews; thematic analysis of focus groups	25	Range: 18–40	25 Orthodox protestant women in the Netherlands	Not reported	Not reported	10 participants were pregnant at time of interview, 15 were not pregnant.	7/10
**16**	2020	Skirrow et al.	England	Mid-wife led antenatal clinic	Influenza and Pertussis	**•** Design: Mixed **•** Approach: IPA **•** Collection: telephone or in-person interview **•** Analysis:	10	Range: 29–44	Not reported	Not reported	Not reported	8 pregnant women at 27–38 weeks’ gestation. 2 postnatal.	7/10
**17**	2020	Gauld et al.	New Zealand	Community	Maternal vaccinations (non-specific)	**•** Design: Qualitative **•** Approach: Framework **•** Collection: Semi- structured interviews **•** Analysis: managed by NVivo Pro.	53	Range: 18–37	9 Māori 4 NZ European 3 South African European 1 Chinese 1 Cook Island	Not reported	Not reported	Women participants were pregnant or had an infant.	8/10
**18**	2022	Arreciado Maranon et al.	Spain	Sexual and reproductive health clinic	Influenza and Pertussis	**•** Design: Qualitative **•** Approach: Not reported **•** Collection: Face-to-face Interviews **•** Analysis: Thematic analysis with ATLAS-ti 8	18	Mean: 33.2 Range: 20–39	Not reported	9 no university degree 9 university degree	Socioeconomic status reported: 9 average 7 below average 2 above average	Women with gestational age range 29–39 weeks	8/10
**19**	2021	Karafillakis et al.	France, Germany, Italy, Spain, UK	Community	Maternal vaccines (e.g., Influenza, pertussis)	**•** Design: Qualitative **•** Approach: Not reported **•** Collection: 20 focus groups and 100 semi- structured interviews **•** Analysis: NVivo thematic analysis	258	Range: 18–46	Not reported	Not reported	Not reported	Pregnant woman at any gestational age	8/10
**20**	2021	Anderson et al.	UK	Community	COVID-19	**•** Design: Qualitative **•** Approach: Not reported **•** Collection: Semi- structured telephone and videoconference interviews **•** Analysis: Thematic analysis manged by NVivo V.11	31	Mean: 33 Range: 24–48	24 White British 1 White European 2 Asian 1 Black 3 Mixed ethnic groups	Not reported	Index of multiple deprivation (IMD): Most frequent 2 or 4	Gestational age ranged 10–39 weeks (mean 24 weeks).	8/10
**21**	2021	Simas et al.	Panama	Community	Maternal vaccines	**•** Design: Qualitative **•** Approach: Not reported **•** Collection: Focus groups and interviews with separate topic guides **•** Analysis: Deductive- inductive thematic analysis, managed by NVivo V.11	56	Mean: 26 Range: 18–39	Not reported	Not reported	Not reported	Pregnant woman at any gestational age	7/10
**22**	2022	Skirrow et al.	UK	Midwife Clinic	COVID-19	**•** Design: Mixed **•** Approach: Not reported **•** Collection: Telephone and face-to-face interviews **•** Analysis: Thematic analysis	10	Range: 25–40	5 White British 1 Black African 1 British Arab 1 British Pakistani 1 White Asian 1 Chinese	Not reported	Not reported	Pregnant women were between 5 to 41weeks’ gestation	7/10
**23**	2022	Ralph et al.	UK	Hospital (tertiary)	Pertussis, influenza and COVID-19	**•** Design: Mixed **•** Approach: Not reported **•** Collection: Semi- structured interviews with follow-up questions. **•** Analysis: Thematic analysis using NVivo	20	Mean: 30.8	13 White 2 Mixed ethnic groups 4 Asian or Asian British 0 Black African Caribbean or Black British 1 Other ethnic group	Not reported	Not reported	Pregnant women at any gestational age; Median gestational age of participants was 26.5 weeks	8/10
**24**	2022	Cooper et al.	USA	Community	Influenza and pertussis	**•** Design: Qualitative **•** Approach: Not reported **•** Collection: Focus groups and semi- structured interview **•** Analysis: Thematic categorisation and visualisation using NVivo 12. Coding by consensus and direct content analysis	18	18 and over	Unknown number of Black women	4 some school 15 school graduate 11 some college 12 university	Not reported	Women who were pregnant at time of interview or pregnant within the past 3 years	7/10
**25**	2022	Gauld et al.	New Zealand	Community	Influenza and pertussis	**•** Design: Qualitative **•** Approach: Not reported **•** Collection: Semi- structured interviews. **•** Analysis: Thematic analysis via NVivo Pro	18	Māori, range: 18–31 Others, range: 23–37	9 Māori 1 Cook Island Māori 4 NZ European 3 South African European 1 Chinese	Not reported	Not reported	Women who were pregnant at time of interview or had a child in the past year	8/10
**26**	2022	Fuss et al.	USA	Hospital Prenatal clinic at a safety-net hospital	Influenza and pertussis (Tdap)	**•** Design: Qualitative **•** Approach: Modified grounded Theory **•** Collection: Individual interviews and brief demographic questionnaire **•** Analysis: Content analysis	28	Mean: 25.3 Range: 18–40	1 Caucasian 15 Black or African American 10 Hispanic/Latinx 1 Haitian 1 Asian	5 some school 9 school graduate 6 some college 5 college graduate 3 graduate school	11 < $20,000 6 $21,000–60,000 3 $61,000–80,000 2 $81,000–100,000 2 >$100,000	Pregnant women at any gestational age	8/10
**27**	2022	Young et al.	New Zealand	Community	Influenza and pertussis	**•** Design: Qualitative **•** Approach: Not reported **•** Collection: Semi- structured interview **•** Analysis: TACT framework used following deductive qualitative content analysis	15	Range: 20–37	9 Māori 1 New Zealander/Māori 3 Samoan 2 Cook Island Māori	Not reported	Not reported	Women who were pregnant at time of interview or had a child in the past year	8/10
**28**	2022	Husain et al.	UK	Hospital	COVID-19	**•** Design: Mixed **•** Approach: Not reported **•** Collection: Structured survey questionnaire **•** Analysis: Descriptive Thematic analysis	441	Mean: 32 Range: 17–44	315 White 77 Asian 9 Black 9 Mixed	Not reported	Not reported	Women who were pregnant at time of survey	7/10

### Barriers and facilitators to vaccine uptake in pregnancy

Five overarching themes emerged from the 28 included studies:

1. perception of disease severity and benefits of vaccination;
2. knowledge, awareness and information sources;
3. vaccine safety, efficacy and trust;
4. healthcare professional interactions;
5. access to vaccination and logistics.

These are summarised in **Table 3**. The first and second-order constructs for the barriers and facilitators to vaccination in pregnancy are presented below.

**Table 3:** Pregnant women’s perceptions of barriers and facilitators to vaccination during pregnancy.

Theme	Barriers	Facilitators	References
Perception of Disease Severity and Benefits of Vaccination	**Perception of low disease severity** • Belief that pregnant women and their infants are ‘low risk’ and not highly susceptible to infection • Seeing the infection affect the mother only and not considering whether it will affect the unborn baby • Perception that influenza is a mild disease, especially in healthy individuals • Preference for ‘natural’ immunity through infection rather than vaccination and preference for ‘natural remedies’ • Perception that personal immunity is sufficient to prevent disease • Belief that good lifestyle habits and personal hygiene guarantee good health• Belief that adults do not need vaccination as much as children do	**Benefits of vaccination and the risk of disease** • Highlighting the benefits of vaccination for the mother and the unborn baby in preventing disease • Seeing pertussis as a disease primarily affecting the foetus (risk to foetus prioritised over risk to mother) • Recognising that infection (e.g., influenza) can lead to severe disease with risks of complications during pregnancy	[68–70, 72–75, 78, 79, 86, 87, 90]
Knowledge, Awareness and Information Sources	**Lack of knowledge** • Lack of awareness that pregnancy is an immune-compromising state with increased infection and complication risks for themselves and the foetus • Lack of knowledge and awareness about vaccines, their necessity, safety and efficacy • Lack of knowledge about when and how to get the vaccines and how and whom it protects **Exposure to misinformation and negative messaging** • Struggling to find reliable sources of information online, overload of non-professional information and feeling overwhelmed • Majority of information on social media about vaccines being negative, casting doubt and causing worry • Exposure to misinformation about vaccines (e.g., vaccination leads to miscarriage and/or autism) • Making decisions based on minimal knowledge	**Knowledge and awareness** •Awareness about the risks of infections in pregnancy, complications and severe disease vaccine-preventable diseases • Aware that these risks can be reduced by vaccination against influenza, pertussis, Covid-19 •Knowledge about the harms caused by pertussis to baby’s health, the desire to avoid hospitalisation and protect the baby **Information sources** •Healthcare professionals, especially doctors, regarded as the most trusted and reliable source of information on vaccination • Receiving adequate and positive information from public health campaigns, news media, antenatal classes and friends • Consistent information about vaccination given throughout the pregnancy	[54, 68, 71–73, 75, 77–88, 91, 92]
Vaccine Safety, Efficacy and Trust	**Concern about vaccination** • Concern and fear about vaccines’ side effects and safety for themselves and their baby • Belief that vaccines are unnecessary and ineffective • Fear of both the vaccine and influenza infection leading to indecision and lack of action by pregnant women • Previous side effects from vaccination and negative experiences of family, friends and peers **Uncertainty and mistrust** • Perceived high risks of vaccination and uncertainty about vaccine safety and efficacy • Unknown risks of vaccination difficult to weigh against potential benefits • Concern about vaccine ingredients (eg., the adjuvant added to the pandemic influenza vaccine) • Perceived insufficient evidence regarding vaccination efficacy and safety • Incompatibility of vaccination with religious beliefs • Fear of being a ‘guinea pig’ and mistrust of government, health systems, healthcare professionals and pharmaceutical companies	**Positive social influences and experiences** Social networks (i.e., family, friends, colleagues) sharing their positive experiences, and encouraging and recommending vaccination • Having had a bad experience with a vaccine-preventable illness personally or through personal network • Having an underlying health condition caused perception of greater risk from illness **Trust and confidence** • Trust in health systems such as hospitals and public health bodies • Confidence and trust in healthcare professionals such as midwives, GPs, obstetricians, pharmacists and health experts knowing what is best for the mother and baby • Consideration that vaccines are God’s gift to keep the baby healthy • Being engaged with healthcare causing to seek out information and preventive measure	[54, 68–75, 77–86, 89–92]
Healthcare Professional (HCP) Interaction	**Lack of effective communication and recommendation** Vaccination not recommended, offered or encouraged by HCP HCP appearing uncertain, hesitant and unclear about vaccination HCP not spending sufficient time to explain, discuss and answer questions on vaccines Not understanding HCP due to language barrier or use of jargon HCP not having the necessary knowledge about vaccine or to deal with its adverse reactions	**Proactive recommendations &clear communication** • Encouragement and recommendation by HCPs particularly midwives, and reassurance about vaccine safety • Clear and consistent messaging delivered with conviction by HCPs endorsing vaccination • Explanation of risks and benefits of vaccination for the mother and baby by a trusted healthcare professional and addressing concerns • HCP having the necessary training and knowledge about vaccines	[68–71, 73–77, 79, 85, 87–89, 93]
Access to vaccination and logistics	**Inconvenience** • Inconvenient vaccination time and location • Having to book an additional appointment just for vaccination (i.e., not offered during routine antenatal care) • Competing priorities and demands during pregnancy leading to a feeling of being overwhelmed and not having time for vaccination • Feeling pressured by HCPs to make a quick decision during a short appointment	**Convenience** • Conveniently located vaccination sites preferably in GP surgery or antenatal clinic during routine visits Workplace vaccination programmes • Availability of influenza, COVID-19 and pertussis vaccines during the same visit	[69, 70, 75, 77, 78, 80, 81, 85–87, 89, 92]

#### Barriers

1. **Perception of Disease Severity and Benefits of Vaccination:** There was a low perception of disease severity for influenza, pertussis and COVID-19 for mothers and babies [68 –71]. Pregnant women often did not see themselves at risk and were unaware of the benefits of vaccinating against these diseases during pregnancy [72, 73]. Some believed their healthy lifestyle habits and knowledge of hygiene were sufficient for maintaining good health [74, 75]. Some women believed in alternative medicine or natural remedies [76, 77]. In one study, higher engagement with their own healthcare increased vaccine refusal [69]. Further, some individuals who had survived a pertussis infection in the past believed that the disease was relatively harmless and not life-threatening, leading them to conclude that vaccination was unnecessary [75, 78]. Similarly, influenza was often not viewed as a serious infection for people in good health and minimised the perceived need for vaccination during pregnancy as an immune-compromising state [69, 74, 75, 79]. Some preferred ‘natural immunity’ from influenza infection and found it difficult to weigh potential benefits against unknown vaccine risks [70]. This mindset extended to beliefs about infant health, with some parents assuming that their baby would receive sufficient antibodies through breastmilk and, therefore, did not require vaccination [75].
2. **Knowledge, Awareness, and Information Sources:** Many pregnant women lacked knowledge and awareness about vaccines’ benefits, necessity, and efficacy [68, 72, 77, 79–85]. Additionally, many were unaware of recommendations for vaccines during pregnancy, [71, 75, 86, 87] and where to receive them [75], leading to decisions based on limited and often incorrect information [85]. In Australia, women from racial minority backgrounds, such as Māori or Pacific Islanders, were notably less aware of pertussis vaccination than white women [88]. Additionally, many women did not receive adequate or convincing information about vaccination from their healthcare providers [87]. Misinformation from online sources (e.g., social media) [75] and peers suggesting that vaccines lead to adverse outcomes such as miscarriages, autism and developmental disorders were significant barriers to vaccination during pregnancy [71, 87, 89]. This issue is compounded by the struggle to find reliable information online, with most vaccine-related content on social media negatively casting doubt and causing worry [90]. Excessive media coverage of the side effects of pandemic influenza vaccine reduced willingness to get vaccinated [79]. Mixed messages and dissuasion from partners or family members also created barriers to vaccination [77, 80].
3. **Vaccine Safety, Efficacy, and Trust:** Concerns over influenza, pertussis and COVID-19 vaccine safety, efficacy, and necessity were prevalent [54, 74, 79, 88, 90]. Women were wary of receiving a COVID-19 vaccine, most perceiving it as riskier than COVID-19 infection [84]. Women cited the unknown safety to them and their babies of a new vaccine as the main reason for personal unwillingness to take a trial COVID-19 vaccine [84]. The risks of the vaccine equalling the risks of COVID-19 were another concern [84]. Concerns about potential side effects of vaccines were significant deterrents, [70, 72, 81, 91], along with the fear of the risk of vaccines in general and [83] to the baby, [71, 73, 75, 80], such as birth defects and autism, [71], especially as mothers cannot see their foetus and detect problems [71]. Other barriers included a belief that women should avoid all medication during pregnancy, fear of being ‘guinea pigs’ for experimental drugs, [71] previous negative experiences with influenza vaccination, [73, 75, 86] influences of family and friends, [74, 77, 87], and perceived risk of infection as a result of receiving the vaccine. [76, 87, 91] There was a view that influenza vaccines may not be effective due to inaccurate predictions of changes in virus strain [75, 87]. Mistrust of doctors, hospitals, and the contents of vaccines and the pharmaceutical industry also contributed [47, 71, 78, 83, 91]. Some Black British Caribbean participants were worried that the government was ‘putting something in people’ through vaccinations and were concerned about the differential effects of vaccines on various demographic groups [78]. Additionally, they felt discriminated against based on ethnicity and socio-economic status [78]. Safety concerns were also cited as barriers, particularly the lack of safety data regarding side effects and effects on the foetus and the speed of COVID-19 vaccine development [47]. The perception that vaccines were against cultural [77] and religious beliefs [82, 89], and concerns about vaccine ingredients were additional barriers [70, 71].
4. **Healthcare Professional Interactions:** The absence of healthcare professional endorsement and offer of vaccination was a notable barrier [68, 76–78]. Concerns included feeling pressured by clinicians to receive vaccination, [70, 71] and to make quick decisions, [91] concerns about one-sided [82], inaccurate and inadequate information [83, 91]. and previously held beliefs [92]. Women also felt a need for better explanations, as they felt that information was either not offered or insufficient [71, 77, 81, 86–88]. Some women felt judged on vaccination and found their concerns dismissed or questions inadequately answered [77, 78]. Sometimes, HCPs delivered information without conviction and the pregnant women had to make autonomous decisions [74]. Poorly informed midwives and GPs’ conflicting advice contributed to confusion about the necessity of vaccines and the appropriate timing for their administration [71]. Another problem was ambiguity among HCPs regarding who should offer the pertussis vaccine—whether it falls under the purview of GPs, obstetricians or midwives [87]. Seeing multiple midwives during pregnancy made building trust and establishing a reliable relationship for advice challenging [78]. There was also an assumption by HCPs that pregnant women were already knowledgeable about pertussis vaccination if they had been pregnant before [81].
5. **Access to Vaccination and Logistics:** Practical concerns such as inconvenient vaccination location and time impeded vaccine uptake, [77, 80] as women juggled vaccination with other competing priorities such as blood tests, scans, and antenatal appointments [69, 81, 86, 92]. Some women also reported challenges like taking time off work, looking after another child and arranging childcare to attend vaccinations [77, 78]. Concerns about onsite safety management around appointment attendance and having to attend a separate appointment for vaccines added to safety concerns, such as the risk of infection at the vaccination setting [84]. Lack of access to vaccinations, also served as a barrier [89]. Women with disruptive life events or other concerns may not prioritise vaccinations, even if pro-vaccination [85]. Some women from marginalised groups, especially non-native English speakers, struggled to understand verbal vaccination information due to accents and medical jargon [78]. Language barriers also led to embarrassment, causing some women to avoid antenatal clinics [78]. Some women were concerned that pharmacies might struggle to handle vaccine side effects and that their busy setting limited discussion about vaccination [93].

#### Facilitators

1. **Perception of Disease Severity and Benefits of Vaccination:** Pregnant women who recognised influenza and pertussis as dangerous conditions, particularly for vulnerable populations or newborns, were more likely to be vaccinated [69, 74–76, 83, 89]. The experience of complications in a previous delivery [74] or heightened concern due to outbreaks like H1N1 in 2009 also contributed to the perceived severity of these diseases [70, 80]. Additionally, pregnant women or their personal networks who previously experienced vaccine-preventable illnesses were more likely to seek vaccination [68, 69, 73–75, 83, 84]. Underlying health conditions caused a perception of greater risk from illness [70]. The perception that maternal immunisation is a social or cultural norm also enhanced vaccine uptake [89] as did highlighting the benefits of vaccination for mother and baby [86]. Some women in France and the UK mentioned community protection, stating that they would consider getting vaccinated to protect those around them [71].
2. **Knowledge, Awareness, and Information Sources:** Women relied on positive personal and family experiences, [74] valuing word-of-mouth from friends and colleagues [74] over media coverage [72]. Vaccinated family members and children at home, and [80] encouragement from social networks (e.g., family, friends and colleagues) also influenced women’s understanding and uptake of vaccines [69, 72, 78, 81, 89]. Women who were knowledgeable about the benefits of vaccination to themselves and their newborns were more likely to accept the vaccine [80]. The more informed women felt, often through consultations with healthcare professionals, the more likely they were to opt for vaccination [92].
  Healthcare providers, primarily doctors and midwives, were regarded as the most reliable and trusted source of information on vaccination [70–72, 82]. Other facilitators included providing concise vaccine information throughout pregnancy, preferably in a wallet-sized pregnancy checklist, [81] receiving positive information from public health campaigns, news media, antenatal classes, and friends, [75, 88] and awareness of and desire to protect against vaccine-preventable diseases for both mother and baby [68, 75, 81, 82, 90]. Consulting religious texts like the Bible, other faith-based resources, and conversations with family, especially spouses, can influence vaccination decisions, [82] as can the perception that vaccines are divinely inspired gifts for children’s health [82].
3. **Vaccine Safety, Efficacy and Trust:** Factors such as market authorisation of vaccines, [74] and trust in healthcare providers such as the UK National Health Service (NHS) contributed to a woman’s confidence in getting vaccinated [47, 69, 77, 81, 83, 84]. Other factors included healthcare professionals offering trustworthy, independent, and unbiased information about vaccine safety, necessity, and effectiveness through brochures and reliable websites, [71, 76, 82, 91] reassurance from healthcare professionals about vaccine safety, [79] and effectiveness in providing immunity to the baby and preventing severe disease and hospitalisation [90].
4. **Healthcare Professional Authorisation:** Healthcare provider (e.g., midwives, obstetricians, GPs) endorsement, recommendation and encouragement facilitated vaccine uptake [47, 68, 69, 71, 73, 76, 77, 79, 80, 83, 87, 88, 92]. Clear, conviction-filled messaging from healthcare professionals, [74, 76], and the explanation of the risks and benefits of the influenza vaccine for the infant were facilitators [71, 72] Positively-framed messaging highlighting the benefits of vaccination was highly preferred [72]. The providers’ ability to listen to pregnant women’s concerns and answer questions were facilitators [71, 81]. Healthcare professionals, especially doctors, were generally seen as reliable and trusted sources of information [54, 73]. Further, pharmacists taking a proactive role in raising awareness of maternal vaccinations, their training to administer vaccines and ongoing relationships with patients were perceived as facilitators [93]. The reputation of the vaccination clinic, and a strong relationship with healthcare professionals were also crucial factors [75].
5. **Access to Vaccination and Logistics:** Conveniently located venues for vaccination, such as GP sites, [80, 85, 87] workplace vaccination programmes, [69] easy and flexible booking systems, and reminder texts improved the likelihood of vaccination [75]. Additionally, when information and vaccines were provided at trusted pharmacies, this was seen as both convenient and accessible [93]. Offering vaccinations in clinics as part of routine antenatal care was important [75].

#### Differences and similarities in attitudes to maternal vaccines

There were differences and similarities between the three recommended vaccines in pregnancy. For pertussis, pregnant women had a more positive attitude about taking the vaccine due to their desire to protect their baby and their perception that children need it more than adults [85, 91]. Women were less aware of vaccine recommendations, efficacy, and necessity for both influenza and pertussis. In contrast, the main barriers to COVID-19 vaccine uptake were concerns about safety and efficacy, given the vaccine’s recent introduction and perceived unknown effects on the baby and future pregnancies [54]. Trusted relationships with healthcare providers, clear and consistent communication of accurate information, convenient access to vaccines, and explanations of vaccine safety, necessity and efficacy were facilitators for all three vaccines.

### Strategies to increase vaccination during pregnancy

**Table 4** summarises the recommendations for addressing barriers and channelling facilitators based on the Five Cs of vaccine hesitancy. The main facilitators are recommendations from a trusted healthcare professional, easy access to vaccination during pregnancy, and clear information on the benefits.

**Table 4:** Key recommendations (Five Cs): addressing participants’ views on barriers and facilitators of vaccine uptake in pregnancy.

Category	Recommendations to increase vaccine uptake among pregnant women: the “five Cs”	References
**1.Confidence** Safety & effectiveness	1. Ensure trusted healthcare professionals (HCP) are providing strong and proactive recommendations, including obstetricians, midwives and general practitioners (GP) who have multiple opportunities to influence pregnant women through longitudinal relationship-based care 2. Encourage empathetic dialogue and tailored communication by trusted HCPs that address women’s concerns and reassure them about vaccination safety and effectiveness 3. Improve confidence by highlighting the scientific rigour, continued research and regulatory monitoring of vaccines, and include pregnant women in future vaccine trials	[54, 68, 73, 76, 78–81, 83–85, 87, 88, 90–92]
**2.Complacency** Perception of risk & disease severity	1. Support pregnant women to understand the risks and benefits of vaccination through their own engagement with trusted information sources 2. Employ effective and empathetic risk communication strategies by trusted HCPs about the increased susceptibility of pregnant women and their babies to severe viral infections	[54, 70, 80, 85, 92]
**3.Convenience** Access barriers	1. Make vaccines and information about them more accessible and deliver vaccination as part of routine antenatal care in primary and secondary care 2. Offer vaccinations in multiple settings, including community pharmacies, general practices midwife-led antenatal clinics and hospitals, with better vaccination reminders and prompts on IT systems (i.e., pop up alerts), 3. Provide convenient appointment times including outside working hours	[69, 71, 73, 75, 77, 78, 80, 83, 85–88, 93, 110]
**4.Communication** Dialogue & relationship	1. Improve communication by healthcare providers and practitioners to focus on the benefits of vaccination, especially for the baby 2. Ensure HCPs (obstetricians GPs and midwives) receive communication training, are knowledgeable about vaccines, maintain a positive attitude toward vaccination, and consistently provide timely, accurate and evidence-based information in various formats during pregnancy 3. Empower pregnant women to evaluate health information on social media. Platforms should exercise more responsibility and accountability by removing misinformed and harmful content. HCPs should be alert to misinformation and rumours and address them appropriately with pregnant women	[72, 74] [68, 69, 71, 76, 77, 79, 81, 89, 90]
**5.Context** Sociodemographic characteristics	1. Engage communities and key groups (i.e., family members, peer networks, community champions and faith leaders) that influence pregnant women in vaccination decision-making 2. Targeted education, awareness and promotion campaigns in multiple languages amongst pregnant women with the lowest vaccine uptake (eg, some ethnic and racial minority groups), and provide support to HCPs to facilitate engagement with a diverse group of people 3. Support equity by identifying and targeting socio-economically vulnerable groups	[68, 71, 78, 82, 86, 91]

Providers who deliver antenatal care, such as obstetricians, midwives and general practitioners, can influence pregnant women to get vaccinated. Pregnant women are more likely to get vaccinated if they perceive the threat of infections during pregnancy, are aware of the benefits and safety of vaccination, and receive a strong recommendation from their healthcare provider. Vaccine accessibility is also crucial in a pregnant woman’s decision to get vaccinated. To increase vaccination uptake, the benefits of vaccination to the infant should be emphasised while also reassuring women about vaccine safety. Educating expectant parents and supporting healthcare professionals with up-to-date knowledge are also crucial in increasing vaccine uptake.

Healthcare providers should take a more proactive role in communication about vaccination during pregnancy, as pregnant women trust them. Translation services should be enhanced, and maternal vaccination leaflets translated into various languages. Access could be improved through standardisation in the organisation of services, and provision of a maternal vaccination helpline. Overall, there is a need for a better approach to vaccination reminders, appointments, and delivery.

## Discussion

### Principal findings

This systematic review identified and synthesised data from a diverse body of primary qualitative research on the barriers and facilitators to vaccine uptake among pregnant women in high-income settings. As described earlier, five overarching themes emerged. These related to personal (i.e., risk perception, knowledge and confidence in vaccines), provider (i.e., healthcare professional attitude and practices) and systemic factors (such as access to and logistics of obtaining the vaccines) during pregnancy. Key barriers were low perceptions of infection risk; lack of knowledge and awareness of vaccine-preventable infections and low perceived need for vaccination, low trust in the safety and effectiveness of vaccines; exposure to misinformation; and poor healthcare professional engagement. Additionally, practical difficulties in obtaining vaccinations were also highlighted by some studies. By contrast, the main facilitators included strong and proactive recommendations by healthcare professionals highlighting the benefits of vaccination, addressing concerns, providing clear, and accurate information throughout pregnancy and positive social influences from family and friends. Easy access to vaccines, such as during routine antenatal care, was also an important facilitator. The key recommendations are summarised in **Table 4**.

### Strengths and limitations

To our knowledge, this is the first systematic review to investigate qualitative studies on barriers and facilitators to vaccination among pregnant women in high-income countries. This was a comprehensive search using 10 databases and the grey literature over the last ten years. We included publications up to and including 2022.

The main limitation is variation in the quality of some of the included data (S2 Table). Many studies did not provide adequate information about the researchers conducting the interviews, their personal biases, or the potential influence of their presence on participants’ responses. Reflexivity was often not considered, which can compromise the validity and reliability of the findings. Some studies did not provide sufficient information about their analytical approach, such as the specific methods used for data analysis or the criteria used to determine themes. This can make it difficult to assess the rigour and quality of the study and limit the ability to compare findings across studies. Additionally, incomplete demographic information (such as educational attainment and income) on participants in the included studies is a limitation, as previous research has demonstrated that vaccine uptake may be lower among those with lower educational attainment and socioeconomic status [94]. While a quarter of studies reported these parameters, the heterogeneity made it difficult to draw meaningful comparisons across the studies.

Another weakness is that the majority of studies were conducted in English-speaking countries (UK, Australia, and the USA), which may not represent the experiences of pregnant women in other cultural contexts and sociodemographic groups. Additionally, some studies had small sample sizes or recruited participants from only one clinical setting, which may not accurately reflect the broader population and may constrain the breadth of perspectives and data saturation. The studies in the qualitative systematic review primarily used interviews, including semi-structured ones. While interviews provide detailed insights, they come with their own set of limitations, such as the potential for interviewer bias, challenges in ensuring data consistency, and limited generalizability due to smaller participant pools. These limitations, however, are not unique to interviews and can similarly impact the reliability and applicability of findings obtained through different research methods. Lastly, the review missed data published outside the specified timeframe or not included in the databases searched.

### Comparison with existing literature

The findings of this study are consistent with recent literature during the COVID-19 pandemic [49, 95, 96]. Further, this study aligns with both our previous qualitative study on vaccine hesitancy among ethnic minorities and our cross-sectional survey of pregnant women in primary care [54, 57]. These factors encompass apprehensions regarding vaccine safety, trust in healthcare professionals as facilitators, and hesitancy towards novel vaccines like COVID-19.

A recent qualitative study also described obstacles like insufficient awareness, mistrust of vaccines and healthcare systems, and suboptimal engagement from healthcare providers in addressing vaccine-related queries [56]. Unequivocal recommendations for vaccination from healthcare providers is crucial [56]. Additionally, belief in myths and conspiracy theories predicts vaccine hesitancy [97]. These beliefs included claims that vaccines are ‘something that the government are putting in people’ [78], the unfounded link between vaccines and autism [87] and that certain vaccines may cause HIV [89].

The COVID-19 pandemic has underscored the importance of trust and the negative impact of unsubstantiated claims and "infodemics" on confidence in vaccines [98–101]. Nevertheless, endorsement of vaccines by trusted healthcare professionals is a powerful predictor of vaccine acceptability, particularly among vulnerable groups [102], which privileges HCPs over other sources of information (e.g. family, friends, colleagues and social media) [94]. This highlights the potential for healthcare professionals, such as midwives, general practitioners, to build trust with patients and help overcome uncertainty and hesitancy [57]. It is worth highlighting that the role of family and friends in vaccine decision-making is multifaceted, potentially swaying individuals both towards and away from vaccination.

### Implications for practice and research

This study provides valuable new insights into the barriers and facilitators of vaccine uptake in pregnancy. The results are highly relevant to the current COVID-19 vaccine deployment and efforts to encourage ‘boosters’ as well as to seasonal influenza and prenatal pertussis vaccinations. Tailored communication and public health campaigns delivered by trusted healthcare professionals are needed to address the concerns of pregnant women. Table 4 provides actionable recommendations from the included studies. Although this review highlights the significance of knowledge and awareness, knowledge alone does not necessarily lead to active behaviour change [103]. Therefore, the role of healthcare professionals is crucial in building confidence and trust.

Supportive environments and tailored messaging are also supported by the Behaviour Change Communication principles [104, 105]. The Capability-Opportunity-Motivation-Behaviour (COM-B) and Theoretical Domains Framework (TDF) are commonly used to guide work on barriers and facilitators to behaviour change [106, 107]. Exposure to misinformation and disinformation shared via social media and informally through family and friends would be very challenging to address [98]. However, clear, consistent, positive vaccine messaging by trusted healthcare professionals and opportunities for open dialogue and discussion, can be helpful. Furthermore, sharing positive vaccination stories, and beliefs, with family, friends and colleagues could be used to channel social influences in the right direction.

Future research should explore the factors specific to certain ethnic and racial minorities, such as Black African and Black Caribbean people, who have the lowest vaccine uptake during pregnancy as well as a higher risk for complications [108]. Furthermore, interventions and strategies to improve vaccine uptake must be backed by rigorous evaluation to determine effectiveness and scalability [109].

## Conclusions

This study provides important insights into the facilitators and barriers to vaccination in pregnancy. These findings can inform the development of targeted interventions to increase vaccine uptake. These should emphasise the importance of healthcare provider recommendations and addressing concerns about vaccine safety and effectiveness. Additionally, future research should explore the facilitators and barriers to vaccine uptake in pregnancy in socioeconomically deprived racial and ethnic minorities to develop interventions tailored to their unique context.

## Supporting information

S1 Table Search strategy. (DOCX)

S2 Table Critical appraisal of included studies (N = 28). (DOCX)

S1 Checklist PRISMA 2020 checklist. (DOCX)
