# Supplementary material for: Facilitators and barriers to vaccination uptake in pregnancy: A qualitative systematic review
Source: PLoS One. 2024 Apr 19;19(4):e0298407. doi: 10.1371/journal.pone.0298407 (PMC11029626; doi:10.1371/journal.pone.0298407)
Supplement: S1 Table — (DOCX) [file pone.0298407.s001.docx]

| **Search strategy:**  **S1 Table: Search stragey**  (((coronavirus or corona-virus) AND (wuhan or beijing or shanghai or Italy or South-Korea or korea or China or Chinese or 2019-nCoV or nCoV or COVID-19 or Covid19 or SARS-CoV* or SARSCov2 or ncov)) OR (pneumonia AND Wuhan) or "COVID-19" or "2019-nCoV" or "SARS-CoV" or SARSCOV2 or 2019-nCov or "2019 coronavirus" or "2019 corona virus" or covid19 or ncov OR "novel corona virus" or "new corona virus" or "nouveau corona virus" or "2019 corona virus" OR "novel coronavirus" or "new coronavirus" or "nouveau coronavirus" or "2019 coronavirus")  OR  Pertussis OR ‘‘whooping cough” OR Pertussis vaccine OR Pertussis vaccin* OR Whooping cough vaccin* OR diphtheria-tetanus-acellular Pertussis Vaccin* OR DTaP Vaccin* OR Diphtheria- Tetanus-Pertussis Vaccin*  OR  flu OR influenza  AND  Vaccin* OR immunis* OR immuniz*  AND  Pregnan*  AND  Strateg* OR intervent* OR campaign* OR evaluat* OR approach OR program*  AND  Hesitan* OR refus* OR accept* OR confidence OR declin* OR reject* OR trust OR distrust OR mistrust OR barrier* OR engag* OR fear OR anxiety OR concern*AND Anxiety OR doubt* OR intent* OR dilemma* OR attitude* OR controvers* OR objector* OR awareness OR dropout* OR perception* OR misconception* OR uptake OR behavi*r OR exemption* OR misinformation OR barrier* OR belief* OR fear* OR opposition OR choice* OR criticis* OR rumo*r OR delay OR mandatory OR compulsory OR knowledge OR decision making OR anti-vaccin* OR parent* con* OR access |
| --- |
