# Supplementary material for: Facilitators and barriers to vaccination uptake in pregnancy: A qualitative systematic review
Source: PLoS One. 2024 Apr 19;19(4):e0298407. doi: 10.1371/journal.pone.0298407 (PMC11029626; doi:10.1371/journal.pone.0298407)
Supplement: S2 Table — (DOCX) [file pone.0298407.s002.docx]

| ID  **S2 Table. Critical Appraisal of Included Studies (N = 28) Abbreviations: Y: Yes, N: No. (**JBI Critical Appraisal Tool for Qualitative Research) | Year | Study Reference | Q1.  congruity between philosophical perspective & methods | Q2.  congruity between methods & research question | Q3.  congruity between methods & data collection | Q4.  congruity between methods & analysis of data | Q5.  congruity between methods & interpretation of results | Q6.  statement locating researcher theoretically | Q7.  influence of researcher on research addressed | Q8.  adequate representation of participants | Q9. evidence of ethical approval | Q10. conclusions drawn from analysis of data |
| --- | --- | --- | --- | --- | --- | --- | --- | --- | --- | --- | --- | --- |
| 1 | 2012 | Schindler et al. | Y | Y | Y | Y | N | N | N | N | Y | Y |
| 2 | 2012 | Meharry et al. | Y | Y | Y | Y | Y | Y | N | Y | Y | Y |
| 3 | 2013 | Marsh et al. | Y | Y | Y | Y | N | N | N | N | Y | Y |
| 4 | 2014 | Collins et al. | Y | Y | Y | Y | Y | N | N | N | Y | Y |
| 5 | 2015 | Donaldson et al. | Y | N | Y | Y | Y | N | N | N | Y | Y |
| 6 | 2015 | Yuen et al. | Y | Y | Y | Y | Y | N | N | Y | Y | Y |
| 7 | 2015 | Wiley et al. | Y | Y | Y | Y | N | Y | Y | N | Y | Y |
| 8 | 2015 | O'Grady et al. | Y | Y | Y | Y | Y | Y | N | Y | Y | Y |
| 9 | 2016 | Winslade et al. | Y | Y | Y | Y | Y | N | N | N | Y | Y |
| 10 | 2016 | Gauld et al. | Y | N | Y | Y | N | Y | Y | N | Y | Y |
| 11 | 2016 | Bettinger et al. | Y | Y | Y | Y | Y | N | N | Y | Y | Y |
| 12 | 2018 | O'Shea et al. | Y | Y | Y | Y | N | Y | Y | N | Y | Y |
| 13 | 2018 | Maisa et al. | Y | Y | Y | Y | Y | N | N | N | Y | Y |
| 14 | 2019 | Wilson et al. | Y | Y | Y | Y | Y | N | N | N | Y | Y |
| 15 | 2020 | de Munter et al. | Y | Y | Y | Y | Y | N | N | N | Y | Y |
| 16 | 2020 | Skirrow et al. | Y | Y | Y | Y | Y | N | N | N | Y | Y |
| 17 | 2020 | Gauld et al. | Y | Y | Y | Y | N | Y | Y | N | Y | Y |
| 18 | 2021 | Maranon et al. | Y | Y | Y | Y | N | Y | Y | N | Y | Y |
| 19 | 2021 | Karafillakis et al. | Y | Y | Y | Y | Y | N | Y | N | Y | Y |
| 20 | 2021 | Anderson et al. | Y | Y | Y | Y | Y | N | N | Y | Y | Y |
| 21 | 2021 | Simas et al. | Y | Y | Y | Y | Y | N | N | N | Y | Y |
| 22 | 2022 | Skirrow et al. | Y | Y | Y | Y | Y | N | N | N | Y | Y |
| 23 | 2022 | Ralph et al. | Y | Y | Y | Y | Y | Y | N | N | Y | Y |
| 24 | 2022 | Cooper et al. | Y | Y | Y | Y | N | Y | N | N | Y | Y |
| 25 | 2022 | Gauld et al. | Y | Y | Y | Y | N | Y | Y | N | Y | Y |
| 26 | 2022 | Fuss et al. | Y | Y | Y | Y | Y | N | N | Y | Y | Y |
| 27 | 2022 | Young et al. | Y | Y | Y | Y | N | Y | Y | N | Y | Y |
| 28 | 2022 | Husain et al. | Y | N | Y | Y | N | Y | Y | N | Y | Y |
